# Early applications of granulocyte colony-stimulating factor (G-CSF) can stabilize the blood–optic-nerve barrier and ameliorate inflammation in a rat model of anterior ischemic optic neuropathy (rAION)

**DOI:** 10.1242/dmm.025999

**Published:** 2016-10-01

**Authors:** Yao-Tseng Wen, Tzu-Lun Huang, Sung-Ping Huang, Chung-Hsing Chang, Rong-Kung Tsai

**Affiliations:** 1 Institute of Eye Research, Buddhist Tzu Chi General Hospital, Hualien, Taiwan; 2 Department of Ophthalmology, Far Eastern Memorial Hospital, Banciao District, New Taipei City, Taiwan; 3 Institute of Medical Sciences, Tzu Chi University, Hualien, Taiwan; 4 Department of Molecular Biology and Human Genetics, Tzu Chi University, Hualien, Taiwan; 5 Department of Dermatology, China Medical University Hospital, Taichung, Taiwan; 6 Department of Dermatology, College of Medicine, China Medical University, Taichung, Taiwan

**Keywords:** Rat anterior ischemic optic neuropathy (rAION), Granulocyte colony-stimulating factor (G-CSF), Blood–optic-nerve barrier, Macrophage infiltration, Microglia/macrophage polarization

## Abstract

Granulocyte colony-stimulating factor (G-CSF) was reported to have a neuroprotective effect in a rat model of anterior ischemic optic neuropathy (rAION model). However, the therapeutic window and anti-inflammatory effects of G-CSF in a rAION model have yet to be elucidated. Thus, this study aimed to determine the therapeutic window of G-CSF and investigate the mechanisms of G-CSF via regulation of optic nerve (ON) inflammation in a rAION model. Rats were treated with G-CSF on day 0, 1, 2 or 7 post-rAION induction for 5 consecutive days, and a control group were treated with phosphate-buffered saline (PBS). Visual function was assessed by flash visual evoked potentials at 4 weeks post-rAION induction. The survival rate and apoptosis of retinal ganglion cells were determined by FluoroGold labeling and TUNEL assay, respectively. ON inflammation was evaluated by staining of ED1 and Iba1, and ON vascular permeability was determined by Evans Blue extravasation. The type of macrophage polarization was evaluated using quantitative real-time PCR (qRT-PCR). The protein levels of TNF-α and IL-1β were analyzed by western blotting. A therapeutic window during which G-CSF could rescue visual function and retinal ganglion cell survival was demonstrated at day 0 and day 1 post-infarct. Macrophage infiltration was reduced by 3.1- and 1.6-fold by G-CSF treatment starting on day 0 and 1 post-rAION induction, respectively, compared with the PBS-treated group (*P*<0.05). This was compatible with 3.3- and 1.7-fold reductions in ON vascular permeability after G-CSF treatment compared with PBS treatment (*P*<0.05). Microglial activation was increased by 3.8- and 3.2-fold in the early (beginning treatment at day 0 or 1) G-CSF-treated group compared with the PBS-treated group (*P*<0.05). Immediate (within 30 mins of infarct) treatment with G-CSF also induced M2 microglia/macrophage activation. The cytokine levels were lower in the group that received immediate G-CSF treatment compared to those in the later G-CSF treatment group (*P*<0.05). Early treatment with G-CSF stabilized the blood–ON barrier to reduce macrophage infiltration and induced M2 microglia/macrophage polarization to decrease the expressions of pro-inflammatory cytokines in this rAION model.

## Abstract (precis)

**Summary:** Early G-CSF treatment (within 24 hours of a rAION infarct) can stabilize optic nerve (ON) vascular permeability to reduce macrophage infiltration into the ON and induce M2 microglia/macrophage polarization at the ON, resulting in decreased inflammation.

## INTRODUCTION

Non-arteritic anterior ischemic optic neuropathy (NAION) is characterized clinically by acute, painless visual loss with optic disc swelling (Hayreh, 2009). NAION is the most common acute optic neuropathy in individuals over 50 years of age, with an estimated annual incidence of 2.3-10.2 per 100,000 people in the United States, and at least 6000 new cases a year (Johnson and Arnold, 1994; Hattenhauer et al., 1997; Hayreh, 2009, 2013). NAION is thought to result from an ischemic insult to the optic nerve (ON) followed by an inflammatory reaction and edema (Salgado et al., 2011; Zhang et al., 2009). Effective treatments for NAION have yet to be established.

The ON head (ONH) is sensitive to blood flow changes and susceptible to vascular insufficiencies caused by altered autoregulation, vasospasm and systemic vascular diseases (Nicholson et al., 2014). Inflammation might be responsible for certain ON damage that occurs in NAION (Hayreh and Zimmerman, 2008; Salgado et al., 2011). In a rat model of anterior ischemic optic neuropathy (rAION), breakdown of the blood–ON barrier was found to occur within hours post-induction of infarct (Bernstein and Miller, 2015), followed by the early recruitment of extrinsic macrophages and activation of resident microglia at the core of the ischemic ON (Slater et al., 2008; Bernstein et al., 2011; Chang et al., 2014; Huang et al., 2015). Activated macrophages have been reported to improve neuronal survival through the secretion of oncomodulin, and to play a key role in phagocytosis and in the removal of myelin debris (soluble Nogo) to enhance remyelination and axon regeneration after ischemic axonal degeneration (Yin et al., 2003; Selvaraju et al., 2004). In addition, the activated M2 phenotype of microglia/macrophages has been reported to have a neuroprotective effect (Orihuela et al., 2016). However, activated microglia/macrophages can also produce harmful substances – such as pro-inflammatory cytokines, proteases and free radicals – that can contribute to neuronal damage after central nervous system (CNS) disorders (Cui et al., 2009). The timing and types of microglia/macrophage activation under different pathological conditions can lead to different actions on retinal ganglion cell (RGC) survival and/or axonal regeneration. Thus, by selective manipulation of inflammatory responses, it might be possible to develop new treatment approaches for NAION.

Granulocyte colony-stimulating factor (G-CSF) is a neuroprotectant that has been used in many ON/retina animal models, including ON axotomy or crush injury, light-induced retinal damage, retinal ischemia and reperfusion, and oxygen-induced retinopathy (Tsai et al., 2008; Oishi et al., 2008; Frank et al., 2009; Shima et al., 2012). We previously demonstrated that G-CSF has a neuroprotective effect in a rat model of anterior ischemic optic neuropathy (rAION model) via the dual actions of anti-apoptosis of RGCs and anti-inflammation on the ON (Chang et al., 2014). A key consideration when evaluating any type of therapy is the timing of treatment; however, it is unclear whether later treatment with G-CSF still has a neuroprotective effect in rAION. In addition, if a therapeutic window exists for the G-CSF treatment of rAION, then it would be interesting to explore the mechanisms contributing to the different results.

**Fig. 1.**
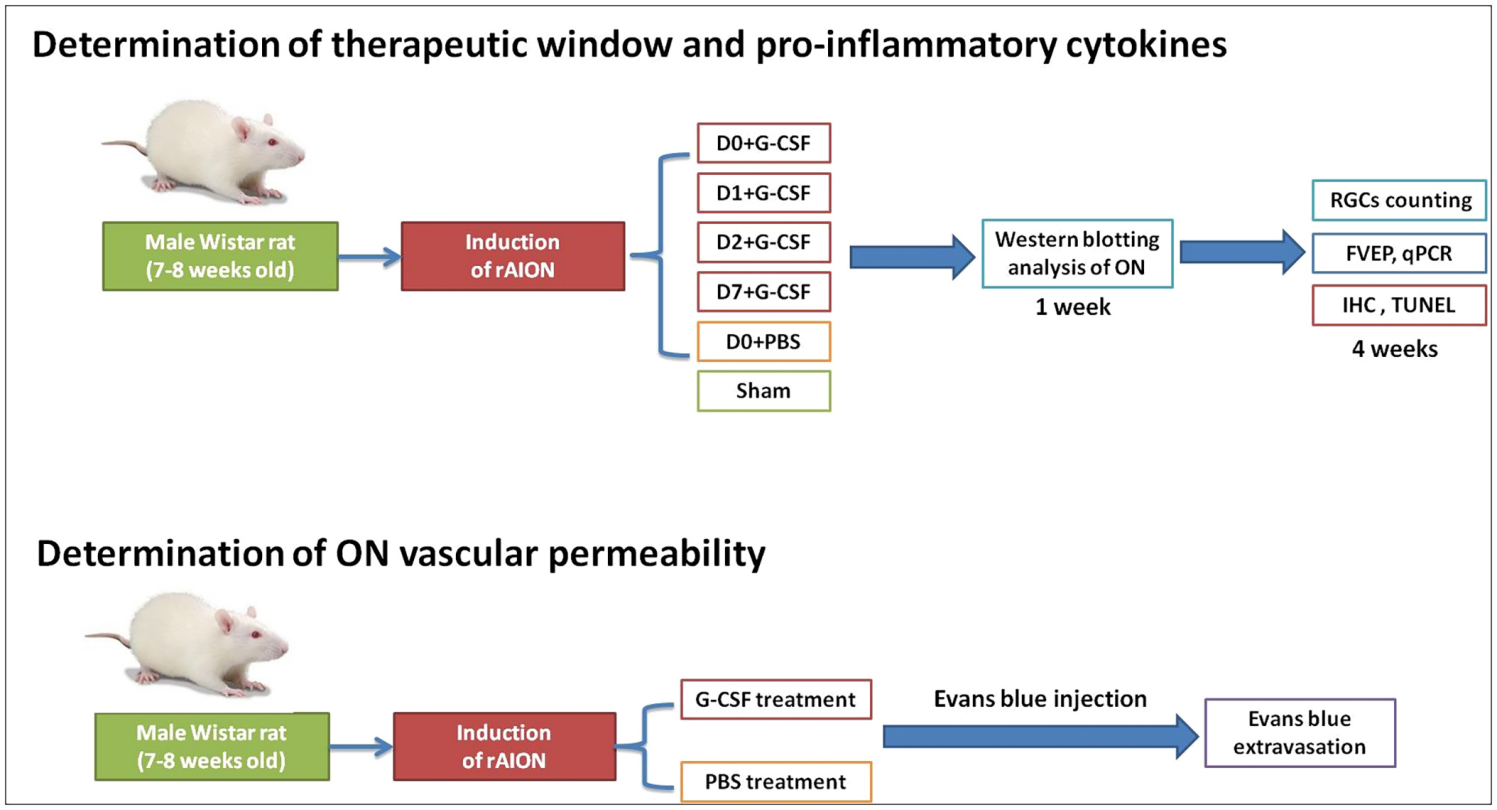
**Illustration of the study design to investigate the therapeutic window of G-CSF treatment and the role of G-CSF in regulating optic nerve (ON) inflammation in a rAION model.** G-CSF, granulocyte colony-stimulating factor; RGCs, retinal ganglion cells; FVEP, flash visual evoked potentials; qPCR, quantitative polymerase chain reaction; IHC, immunohistochemistry; TUNEL, *in situ* nick end-labeling.

Ischemic-brain-injury models have demonstrated that G-CSF treatment can reduce neuroinflammation, neutrophil infiltration, infarct volume and blood–brain barrier (BBB) disruption (Lee et al., 2005; Doycheva et al., 2014; Li et al., 2015). Evidence from rAION models suggests that reducing inflammation and ON edema in the acute phase may be effective in protecting neurons from degeneration (Goldenberg-Cohen et al., 2005; Slater et al., 2008; Zhang et al., 2009, 2010; Touitou et al., 2013; Chang et al., 2014; Lee et al., 2014). Therefore, it is important to identify the optimal timing of G-CSF treatment in rAION and the related mechanisms. Herein, the purpose of this study was to determine the therapeutic window of G-CSF in a rAION model and to investigate the mechanisms by which G-CSF exerts an anti-inflammatory effect on an ON (Fig. 1).

## RESULTS

### Early G-CSF treatments (before 24 h) post-infarction was beneficial in preserving visual function

**Fig. 2.**
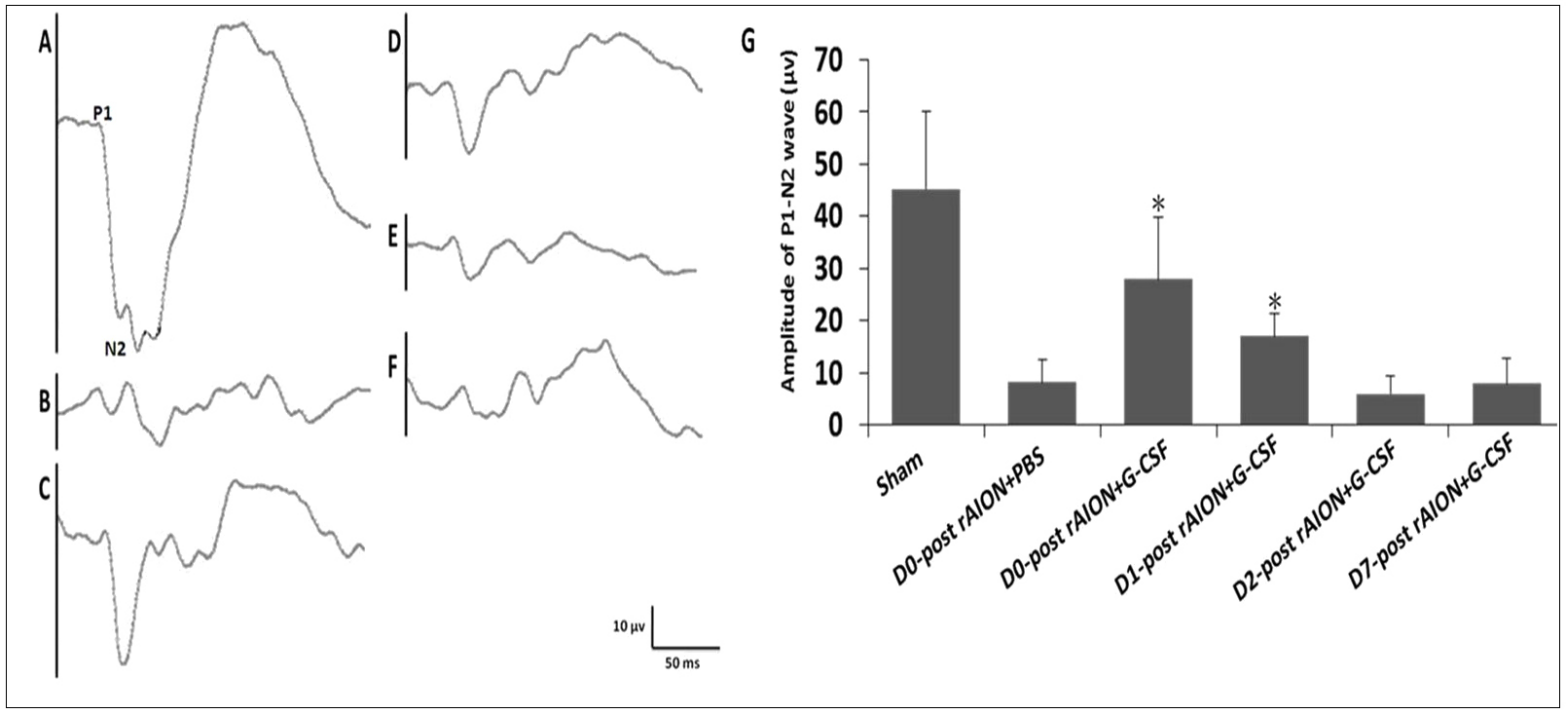
**Evaluation of the recovery of injured optic nerves (ONs) by FVEPs in the rAION model.** Representative FVEP tracings at 4 weeks following rAION induction in (A) sham, (B) D0+PBS, (C) D0+G-CSF, (D) D1+G-CSF, (E) D2+G-CSF and (F) D7+G-CSF groups. (G) Bar charts showing P1-N2 amplitude. Data are expressed as mean±s.d. in each group (*n*=6 in each group). The amplitude of the P1-N2 waves in the D0+G-CSF-treated group and D1+G-CSF-treated group were significantly higher than that of the PBS-treated group [27.9±11.9 μV (*P*=0.005) and 16.9±4.4 μV (*P*=0.013) vs 8.1±4.3 μV, respectively]. *P<0.05 compared with D0 PBS treatment by the Mann–Whitney *U*-test.

The latency of the P1 wave was consistently 34.9±3.6 ms among the groups in the flash visual evoked potential (FVEP) tests. The amplitudes of the P1-N2 waves in the day 0 (D0)+G-CSF- and D1+G-CSF-treated groups were significantly higher than that of the PBS-treated group [27.9±11.9 μV (*P*=0.005) and 16.9±4.4 μV (*P*=0.013) vs 8.1±4.3 μV, respectively] (Fig. 2).

### Early (before 24 h post-infarction) G-CSF treatments preserved the survival and reduced the apoptosis of RGCs

**Fig. 3.**
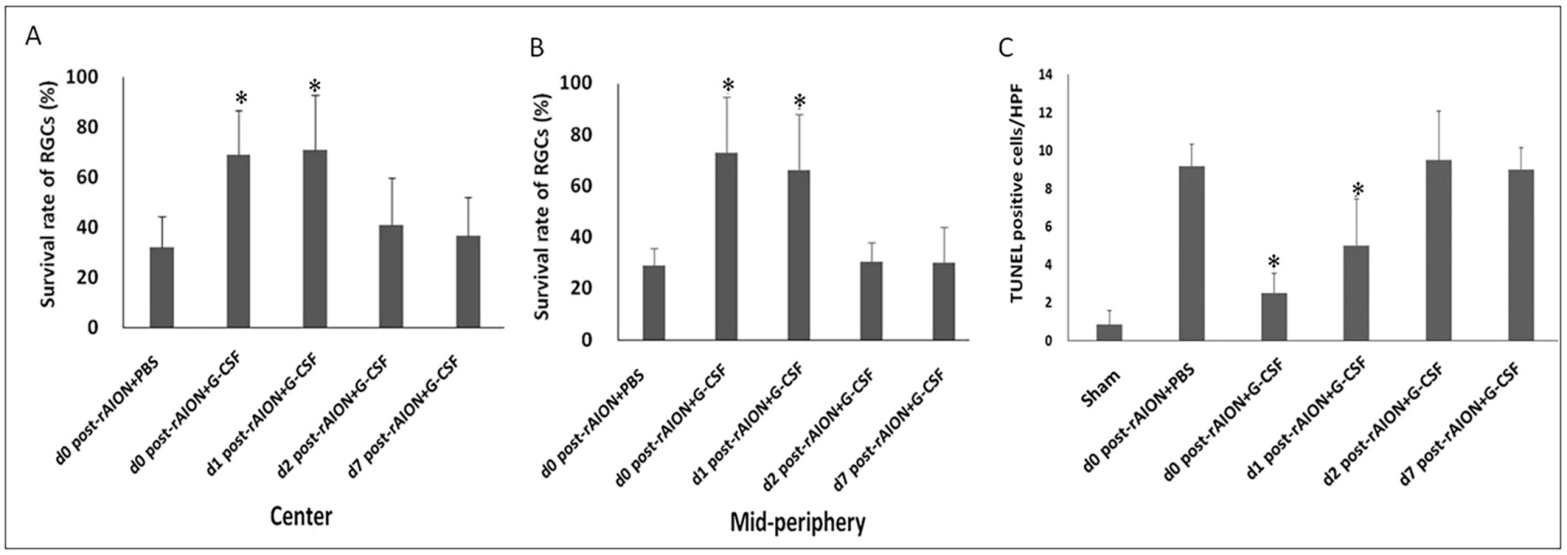
**Survival rate of RGCs and apoptotic RGCs in rAION-induced rats with PBS treatment and G-CSF treatment on day 0, 1, 2 or 7 post-rAION induction.** (A) The RGC survival rates of central retinas and (B) mid-peripheral retinas in each group were calculated as a percentage of the RGC density in the sham group. (C) Quantification of TUNEL-positive cells per high-power field (HPF). Data are expressed as mean±s.d. in each group (*n*=6). In the central retinas, the survival rates of the RGCs in the D0+G-CSF-treated group and D1+G-CSF-treated group were both 2.2-fold higher than that of the PBS-treated group (*P*=0.013 and *P*=0.020, respectively). In mid-peripheral retinas, the RGC survival rates in the D0+G-CSF-treated group and D1+G-CSF-treated group were 2.5-fold (*P*=0.005) and 2.3-fold (*P*=0.005) higher compared to the PBS-treated group. The numbers of TUNEL-positive cells/HPF in the D0+G-CSF-treated group and D1+G-CSF-treated group were 3.7- and 2.0-fold lower than that of the PBS-treated group, respectively (*P*=0.005, *P*=0.013). *P<0.05 compared with D0 PBS treatment by the Mann–Whitney *U*-test.

G-CSF treatment starting at day 0 and day 1 post-rAION induction preserved a higher density of RGCs both in central and mid-peripheral retinas compared to treatment starting at day 2 and day 7 post-rAION induction (Fig. 3). The survival rates of the RGCs in central retinas were 32.0% in the PBS-treated group, 68.8% in the D0+G-CSF-treated group, 71.0% in the D1+G-CSF-treated group, 40.8% in the D2+G-CSF-treated group and 36.5% in the D7G-CSF-treated group (Fig. 3A). In addition, the survival rates of the RGCs in the D0+G-CSF- and D1+G-CSF-treated groups were both 2.2-fold higher than that of the PBS-treated group (*P*=0.013 and *P*=0.020, respectively). In mid-peripheral retinas, the RGC survival rates were 28.8% in the PBS-treated group, 73.1% in the D0+G-CSF-treated group, 66.4% in the D1+G-CSF-treated group, 30.5% in the D2+G-CSF-treated group and 30.2% in the D7+G-CSF-treated group (Fig. 3B). The survival rates of the RGCs in the mid-peripheral retinas in the D0+G-CSF- and D1+G-CSF-treated groups were 2.5-fold (*P*=0.005) and 2.3-fold (*P*=0.005) higher, respectively, compared to the PBS-treated group (Fig. 3B).

The number of TUNEL-positive cells per high-powered field (cells/HPF) in the RGC layers of the retinas were 0.83±0.75 in the sham-operated group, 9.17±1.17 in the PBS-treated group, 2.50±1.05 in the D0+G-CSF-treated group, 4.6±2.3 in the D1+G-CSF-treated group, 9.50±2.59 in the D2+G-CSF-treated group and 9.00±1.00 in the D7+G-CSF-treated group (Fig. 3C). The numbers of TUNEL-positive cells/HPF in the D0+G-CSF- and D1+G-CSF-treated groups were 3.7- and 2.0-fold lower than that of the PBS-treated group, respectively (*P*=0.005, *P*=0.013) (Fig. 3C).

### Early (before 24 h post-infarction) G-CSF treatments reduced the infiltration of extrinsic vascular-borne macrophages in ONs

**Fig. 4.**
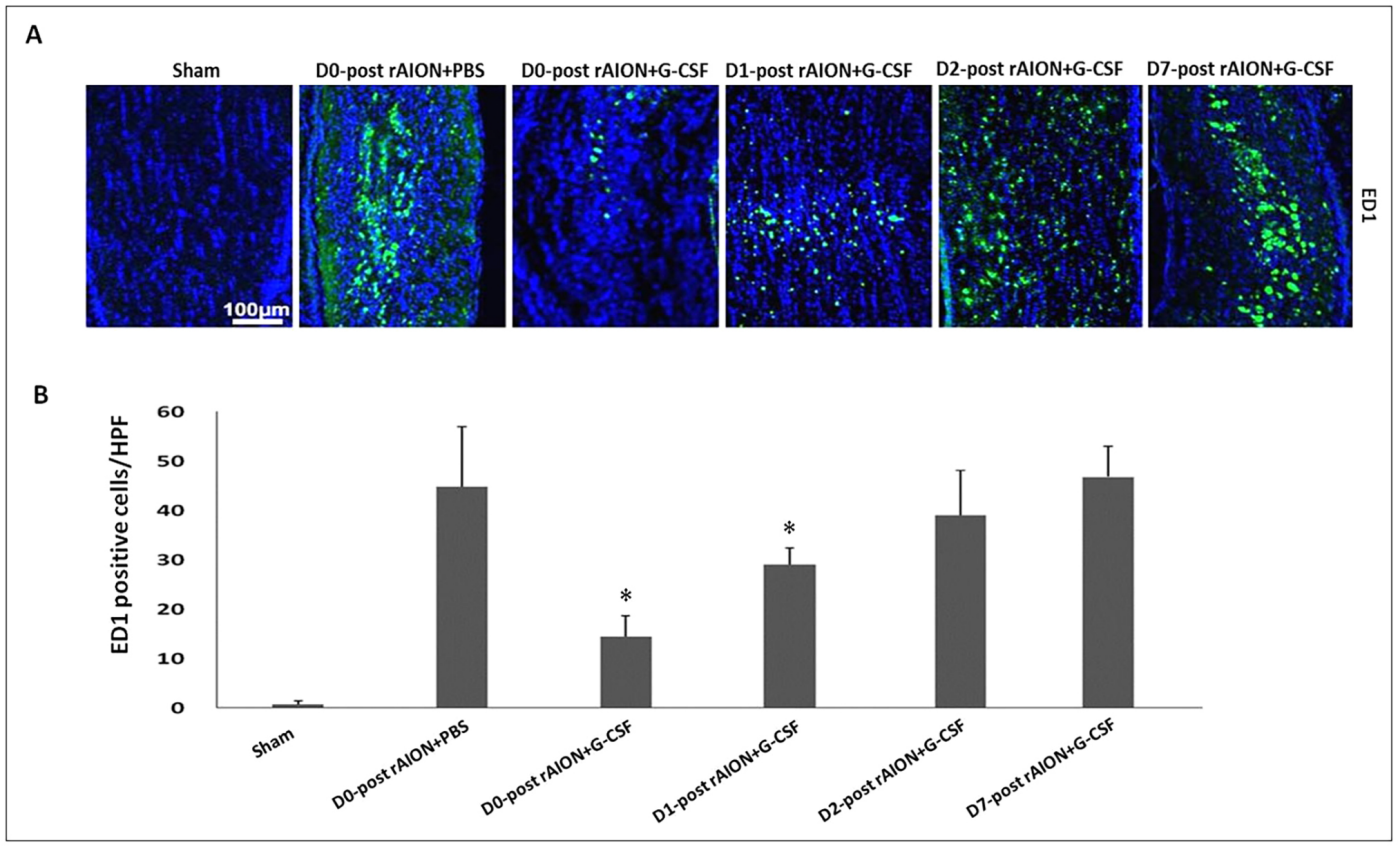
**Immunohistochemistry of ED1 in optic nerves (ONs) at 4 weeks after rAION induction to evaluate macrophage infiltration.** (A) The representative figures show anti-ED1 staining in longitudinal sections of ONs. The ED1-positive cells in green were labeled with FITC, and the nuclei in blue were stained with DAPI. (B) Quantification of ED1-positive cells per high-power field (HPF). Data are expressed as mean±s.d. in each group (*n*=6 in each group). The level of extrinsic macrophage infiltration was reduced by 3.1- and 1.6-fold by G-CSF treatment starting on day 0 and day 1 post-rAION induction, respectively, compared to PBS treatment (*P*=0.005, *P*=0.013). *P<0.05 compared with D0 PBS treatment by the Mann–Whitney *U*-test.

The cells on the ONs were stained with DAPI, whereas the newly synthesized (extrinsic) macrophages were stained with anti-ED1 (Fig. 4A). Quantification analysis showed 0.67±0.82 ED1-positive cells/HPF in the sham-operated group, 44.83±12.19 ED1-positive cells/HPF in the PBS-treated group, 14.50±4.18 ED1-positive cells/HPF in the D0+G-CSF-treated group, 28.00±3.67 ED1-positive cells/HPF in the D1+G-CSF-treated group, 46.80±6.22 ED1-positive cells/HPF in the D2+G-CSF-treated group and 43.60±9.34 ED1-positive cells/HPF in the D7+G-CSF-treated group (Fig. 4B). The level of extrinsic macrophage infiltration was reduced by 3.1- and 1.6-fold by G-CSF treatment starting on day 0 and day 1 post-rAION induction, respectively, compared to PBS treatment (*P*=0.005, *P*=0.013) (Fig. 4B).

### Early (before 24 h post-infarction) G-CSF treatments increased the infiltration of intrinsic microglia in ON

**Fig. 5.**
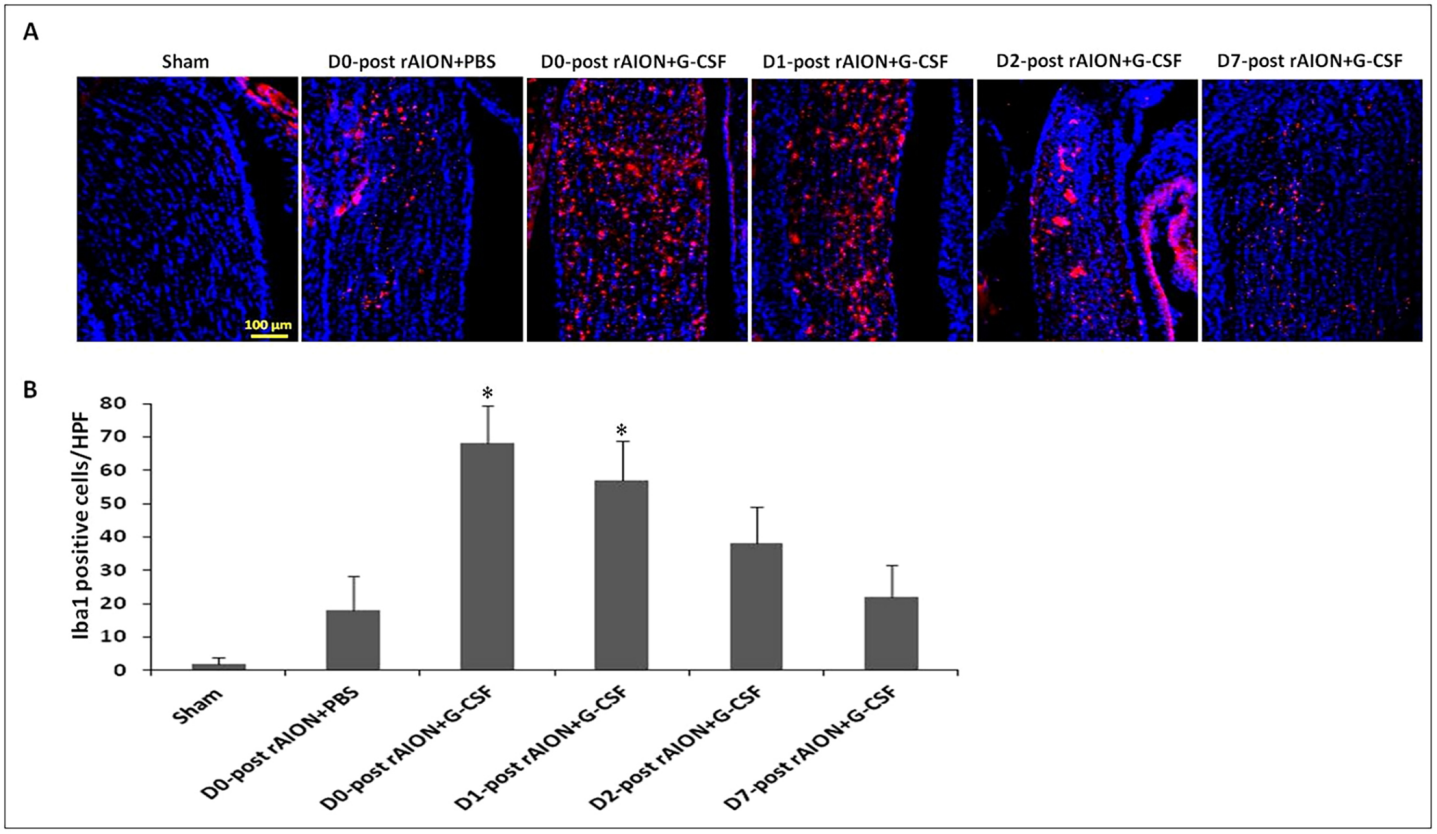
**Immunohistochemistry** **of Iba1 in optic nerves (ONs) at 4 weeks after rAION induction to evaluate microglial activation.** (A) The representative figures show anti-Iba1 staining in longitudinal sections of ONs. The Iba1-positive cells in red were labeled with Rhodamine, and the nuclei in blue were stained with DAPI. (B) Quantification of Iba1-positive cells per high-power field (HPF). Data are expressed as mean±s.d. in each group (*n*=6 in each group). The levels of intrinsic microglia activation were increased by 3.8- and 3.2-fold by G-CSF treatment starting on day 0 and 1 post-rAION induction compared to PBS treatment (*P*=0.005, *P*=0.005). *P<0.05 compared with D0 PBS treatment by the Mann–Whitney *U*-test.

The intrinsic microglia were stained with anti-Iba1 (Fig. 5A). Quantification analysis showed 2.12±1.82 Iba1-positive cells/HPF in the sham-operated group, 18.38±10.19 Iba1-positive cells/HPF in the PBS-treated group, 68.30±11.28 Iba1-positive cells/HPF in the D0+G-CSF-treated group, 57.06±11.71 Iba1-positive cells/HPF in the D1+G-CSF-treated group, 38.08±10.92 Iba1-positive cells/HPF in the D2+G-CSF-treated group and 22.18±9.43 Iba1-positive cells/HPF in the D7+G-CSF-treated group (Fig. 5B). The level of intrinsic microglia activation was increased by 3.8- and 3.2-fold by G-CSF treatment starting on day 0 and 1 post-rAION induction, respectively, compared to PBS treatment (*P*=0.005, *P*=0.005) (Fig. 5B).

### Early (before 24 h post-infarction) G-CSF treatments stabilized the vascular permeability of the ONs: the blood–ON barrier

**Fig. 6.**
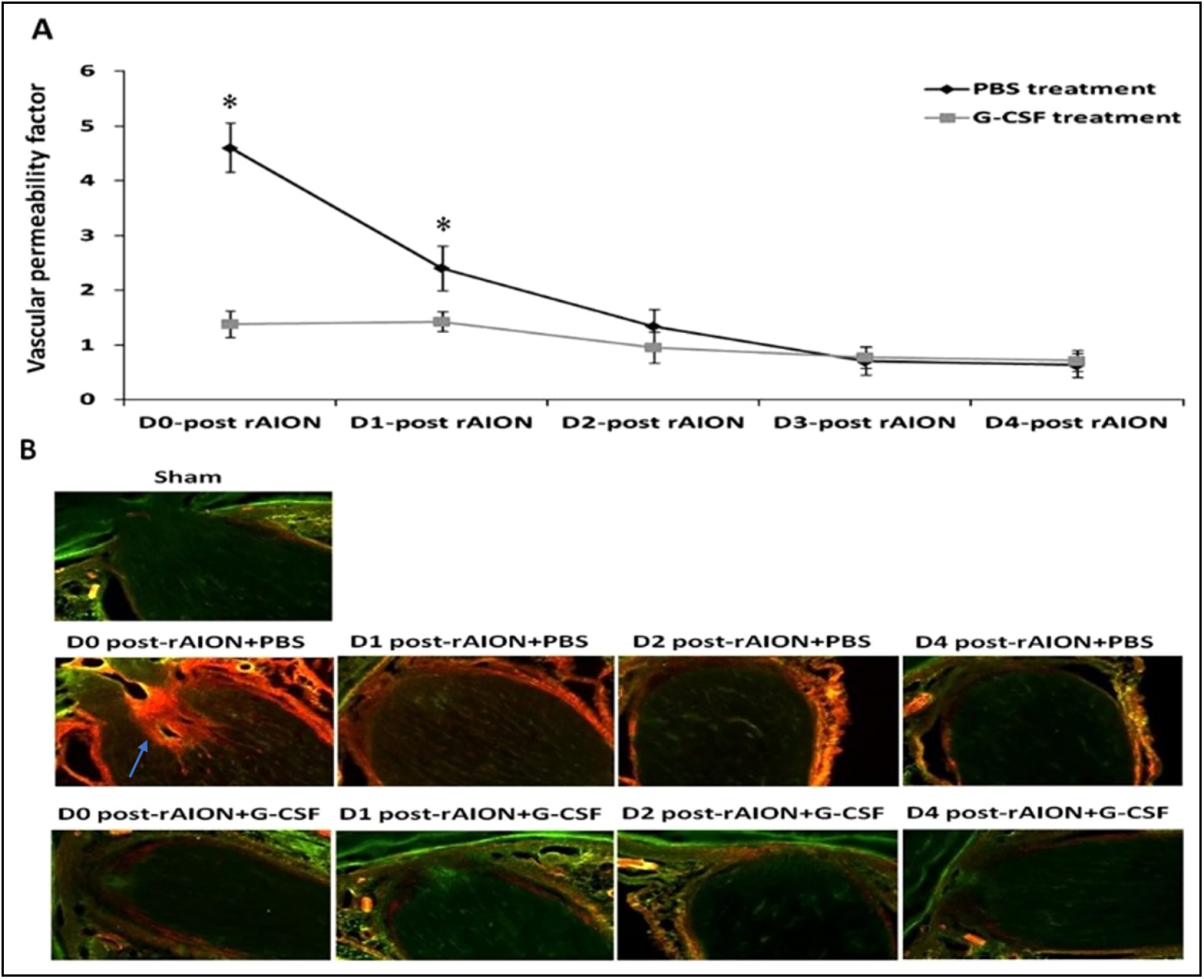
**Optic nerve (ON) vascular leakage in the PBS-treated rats and the G-CSF-treated rats.** (A) Evans-Blue-based quantification of ON vascular permeability. G-CSF treatment starting on day 0 and day 1 post-rAION induction reduced ON vascular permeability by 3.3- and 1.7-fold compared to PBS treatment (*P*<0.05). Data are expressed as mean±s.d. in each group (*n*=6 in each group). *P<0.05 by the Mann–Whitney *U*-test. (B) Evans Blue fluorescence image of ON sections. The Evans-Blue-stained ON tissue emitted orange red fluorescence (blue arrow) and the unstained ON tissue emitted green auto-fluorescence. G-CSF treatment starting on day 0 and day 1 post-rAION induction reduced Evans Blue leakage in the ON sections compared with PBS treatment.

The Evans Blue permeability experiments showed that the vascular permeability of the blood–ON barrier was disrupted immediately after rAION induction, and then gradually recovered 2-3 days post-infarct in the PBS-treated ONs. The G-CSF treatment starting on day 0 and day 1 post-rAION induction significantly reduced ON vascular permeability by 3.3- and 1.7-fold, respectively, compared to PBS treatment (*P*<0.05) (Fig. 6A). Evans Blue leaked through the disrupted blood–ON barrier and infiltrated the ON post-infarct. The Evans-Blue-stained tissue emitted orange red fluorescence, and the unstained ON tissue emitted green auto-fluorescence (Fig. 6B). Fig. 5B shows that G-CSF treatment starting on day 0 and day 1 post-rAION induction reduced the Evans Blue leakage in the ON sections compared with PBS treatment. The results of Evans Blue extravasation and fluorescence demonstrated that early treatment with G-CSF reduced blood–ON barrier disruption in the rAION model, which was compatible with the time course of macrophage infiltration at the ONs post-infarction.

### Immediate (within 30 min post-infarction) G-CSF treatment induced the expression of M2 microglia/macrophage markers

**Fig. 7.**
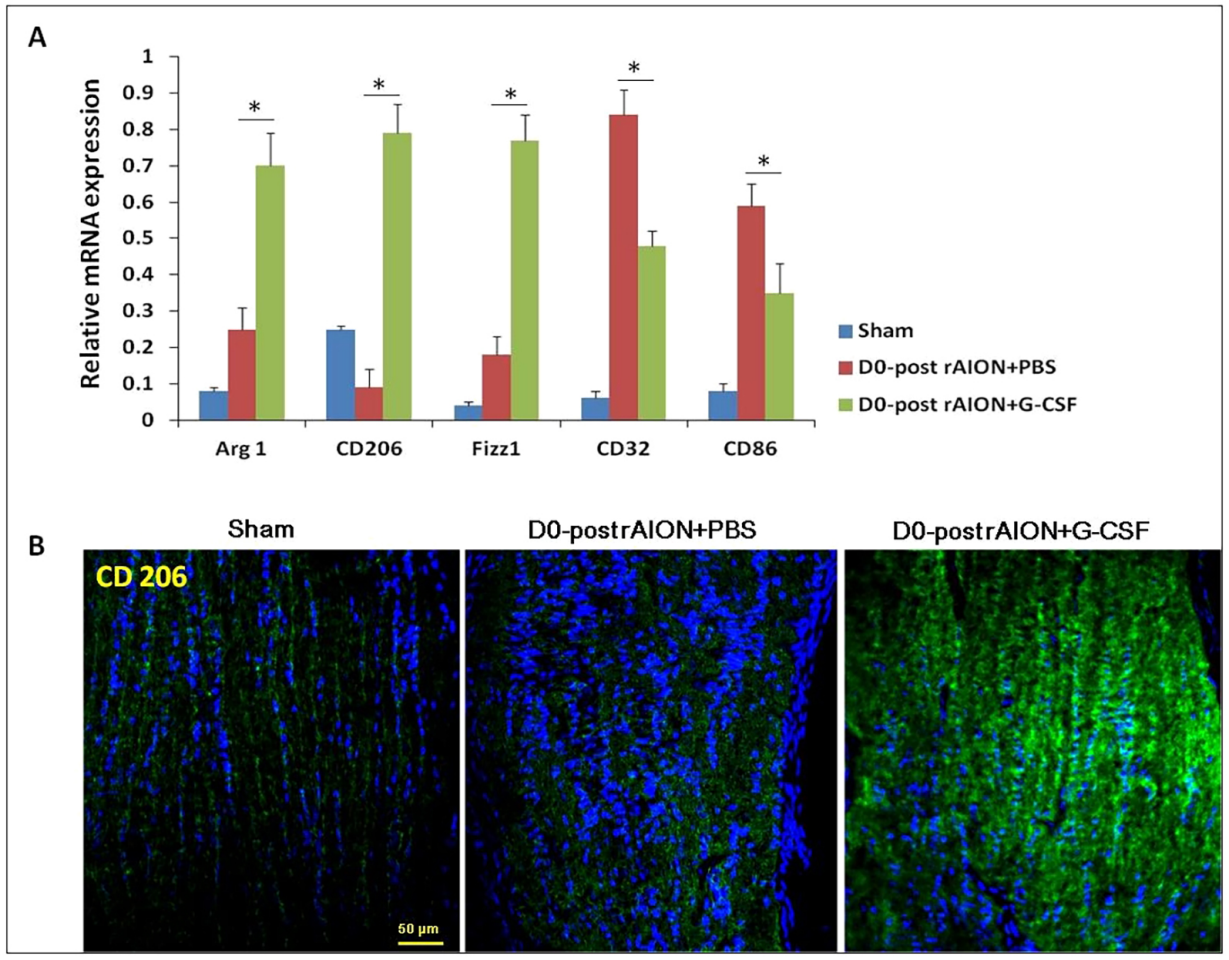
**Relative mRNA expression levels of markers of M1 and M2 macrophages in the optic nerves (ONs) are shown as histograms.** (A) Each value was normalized to *GypA*. Statistical analysis indicated that the expressions of *Arg1*, *CD206* and *Fizz1* (markers of M2 macrophages) increased after immediate treatment with G-CSF compared to treatment with PBS (*P*<0.05). *CD32* and *CD86* (markers of M1 macrophages) decreased after immediate treatment with G-CSF compared to treatment with PBS (*P*<0.05). *P<0.05 by the Mann–Whitney *U*-test. (B) Representative CD206 IHC stainings of ONs. Note that CD206-positive cells increased in the ON of D0 post-rAION+G-CSF rats as compared with that of sham and PBS-treated rats.

Immediate G-CSF treatment [within 30 min post-infarct (D0+G-CSF)] induced the highest level of microglial infiltration in the ONs (Fig. 5B). To further characterize the microglia/macrophage phenotype, we measured the representative genes of M1/M2 macrophages using quantitative real-time PCR (qRT-PCR) analysis. The results showed that expressions of Arg1, CD206 and Fizz1 (markers of M2 macrophages) were increased, whereas expressions of CD32 and CD86 (markers of M1 macrophages) were decreased in the ONs after immediate treatment with G-CSF compared to PBS treatment (Fig. 7).

### Immediate G-CSF treatment post-infarction significantly reduced pro-inflammatory cytokine production in the damaged ONs

**Fig. 8.**
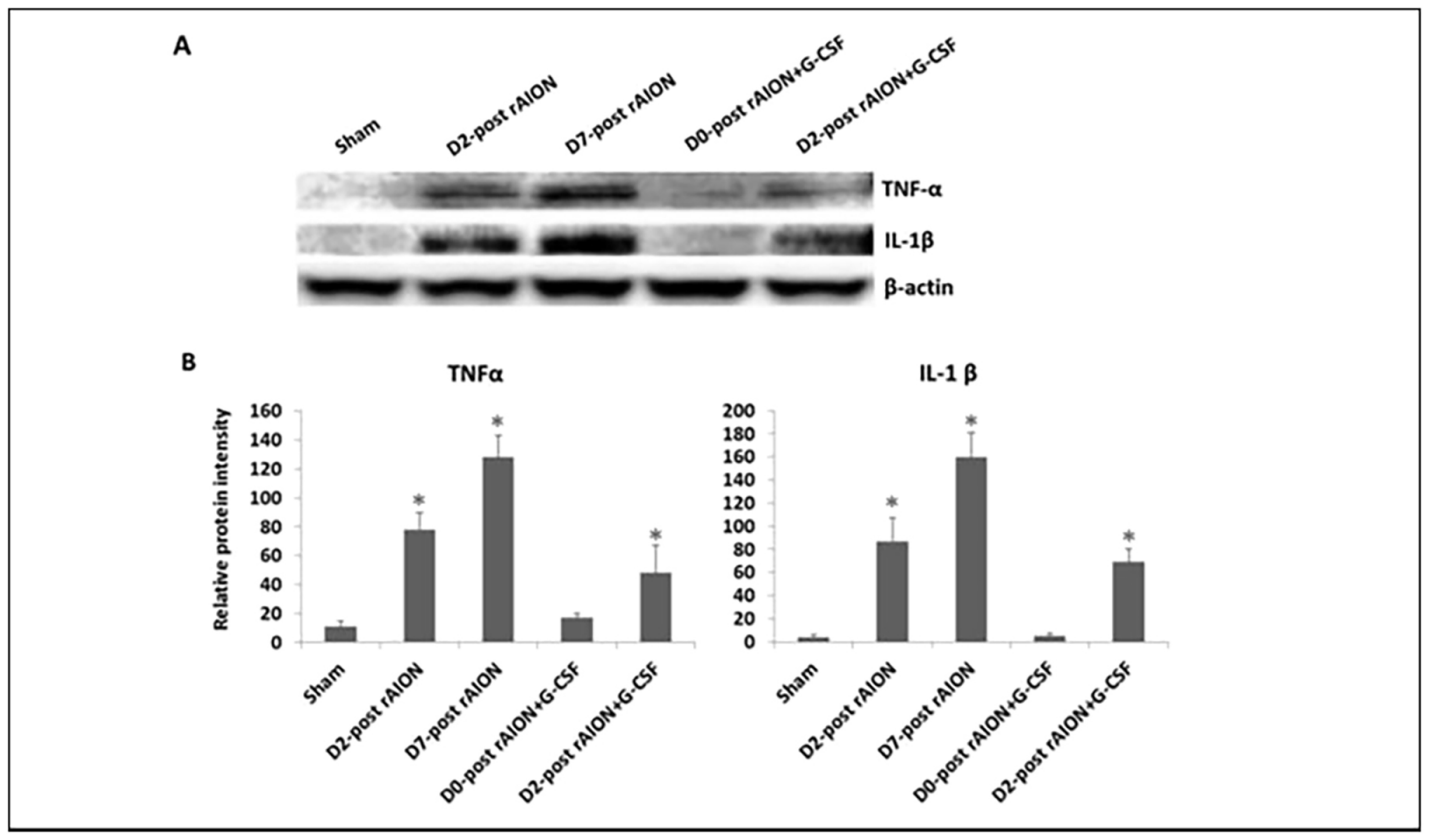
**The expressions of TNF-α and IL-1β in the optic nerve (ON) tissues.** (A) Analysis of TNF-α and IL-1β expressions using western blotting. (B) Quantification of the protein bands of TNF-α and IL-1β. Data are expressed as mean±s.d. in each group (*n*=6 in each group). The TNF-α expression in the ON tissues was increased by 7.1- and 11.6-fold after 2 and 7 days post-rAION induction, respectively, compared to the sham controls (*P*<0.05). The IL-1β level in the ON tissues was increased by 21.8- and 40.8-fold after 2 and 7 days post-rAION induction, respectively, compared to the sham controls (*P*<0.05). *P<0.05 compared with the sham control by the Mann–Whitney *U*-test.

To determine the levels of pro-inflammatory cytokines in the ON tissues, the protein expressions of TNF-α and IL-1β in the ON samples were evaluated by western blotting analysis (Fig. 8A). TNF-α expression in the ON tissues was increased by 7.1- and 11.6-fold after 2 and 7 days post-rAION induction, respectively, compared to the sham controls (*P*<0.05) (Fig. 8B). In addition, G-CSF treatment starting on day 2 post-rAION induction increased the TNF-α expression in the ON tissues by 4.4-fold compared with the sham controls (*P*<0.05) (Fig. 8B). The IL-1β level in the ON tissues was increased by 21.8- and 40.8-fold after 2 and 7 days post-rAION induction, respectively, compared to the sham controls (*P*<0.05) (Fig. 8B). G-CSF treatment starting on day 2 post-rAION induction increased the IL-1β expression by 17.3-fold in the ON tissues compared to the sham controls (*P*<0.05) (Fig. 8B). However, there were no significant differences in the levels of TNF-α and IL-1β between G-CSF treatment starting on day 0 post-rAION induction and the sham controls (Fig. 8B).

## DISCUSSION

In the present study, we demonstrated that the time post-infarct of initiating treatment with G-CSF decided the outcome of visual function and morphometry of RGC preservation in rAION. Specifically, early treatment (before 24 h) resulted in better visual function than delayed treatment, as demonstrated by better preservation of amplitudes in FVEPs, and increased survival of RGCs. These findings clearly show that a therapeutic window exists in G-CSF treatment of rAION (before 1 day post-infarct). We further clarified the mechanisms by which G-CSF regulates neuroinflammation. In a rAION model, the blood–ON barrier is disrupted soon after the infarct, allowing for vascular-borne macrophages to cross the barrier and infiltrate into the ON with the massive production of TNF-α and IL-1b, which may then cause further damage to neurons. In this study, immediate subcutaneous G-CSF treatment restored the disrupted blood–ON barrier and prevented extrinsic macrophage infiltration. In addition, it induced M2-type microglia/macrophage activation in the damaged ON, which effectively reduced the production of TNF-α and IL-1β in the damaged ON, and maintained the RGCs in an anti-apoptotic environment.

Inflammation is a prominent histological feature of rAION, and both ED1-positive and Iba1-positive cells have been reported to immediately surround anterior ONs 1 day post-infarct (Zhang et al., 2009). Furthermore, Zhang et al. reported marked infiltration and aggregation of ED1-positive and Iba1-positive cells 3 days post-infarct, with axon disruption in the region of the ischemic infarct (Zhang et al., 2009). This suggests that specific modulation of inflammation at an early stage may be a useful approach for NAION treatment (Zhang et al., 2009; Bernstein et al., 2011; Salgado et al., 2011). Our results demonstrated that G-CSF can modulate neuroinflammation of the infarcted ON during a specific therapeutic window (before 24 h), and this window is closely associated with the restoration of the disrupted blood–ON barrier. In addition, attempting to stabilize the blood–ON barrier with G-CSF treatment after 2 days post-infarct proved to be too late, because most extrinsic macrophages had already migrated into the ON before 2 days post-rAION induction. Lee et al. reported that G-CSF reduced blood permeability and inflammatory infiltration in a rat ischemic brain model (Lee et al., 2005). It has been reported that G-CSF attenuates BBB disruption and neuroinflammation in neonatal rat models of hypoxia-ischemia (Doycheva et al., 2014; Li et al., 2015). In a damaged CNS in which the BBB is broken, activated macrophages are derived mainly from blood-borne recruited cells (Giulian et al., 1989). The activated macrophages accumulate in the injured CNS and change the local microenvironment through the production of inflammatory cytokines such as TNF-α, IL-1 and IL-6 (Leskovar et al., 2000). TNF-α is toxic to oligodendrocytes and RGCs (Nakazawa et al., 2006). Our results also showed that early treatment with G-CSF decreased macrophage infiltration and the expression of inflammatory cytokines at the infarcted ON. Taken together, we suggest that a potential mechanism by which early treatment with G-CSF (before 1 day post-infarct) attenuates blood–ON barrier disruption in rAION is by reducing macrophage infiltration and pro-inflammatory-cytokine-induced injury.

Zhang et al. reported that Iba1-positive cells in the intrascleral and immediate retroscleral portions of the ON largely disappear by 14 days post-rAION induction (Zhang et al., 2009). As expected, we found few Iba1-positive cells on the ON at the fourth week post-infarct in the PBS-treated group and the groups that started G-CSF treatment on days 2 and 7 post-infarct. Early treatment with G-CSF induced more Iba1-postive cells on the ON 4 weeks after rAION induction. A possible reason for this is that the majority of intrinsic microglia are activated to remove myelin debris when there is a lack of extrinsic macrophage infiltration in the ONs. Microglia are crucial nervous-system-specific immune cells that serve as tissue-resident macrophages. Microglia/macrophages can be induced by lipopolysaccharide (LPS) or interferon-γ stimulations to an M1 phenotype for the expression of pro-inflammatory cytokines or by IL-4/IL-13 stimulation to an M2 phenotype for tissue repair and resolution of inflammation (Orihuela et al., 2016). Interestingly, we found that immediate treatment with G-CSF promoted M2 polarization of microglia/macrophages post-ON infarct in the rAION model. M2 microglia have been reported to exert a neuroprotective effect (Orihuela et al., 2016). Our western blotting results showed that the levels of pro-inflammatory cytokines decreased in the early G-CSF-treated groups. Thus, early treatments with G-CSF may also regulate the microglia/macrophage phenotype to reduce proinflammatory cytokines on the ON in a rAION model. G-CSF can selectively activate STAT3 in human monocytes to inhibit LPS-induced pro-inflammatory cytokine production (Nishiki et al., 2004). Taken together, we believe that G-CSF may reduce pro-inflammatory cytokine production in a rAION model via the alternation of microglia/macrophage activation and direct activation of monocytes. Our results support the idea that early treatment with G-CSF can increase the number of activated microglia and induce M2 microglia/macrophage polarization at the ON, which may result in a neuroprotective effect in a rAION model.

Anti-apoptotic effects of G-CSF have been reported in CNS models and RGCs (Hartung et al., 1995; Solaroglu et al., 2007; Zhao et al., 2011; Kadota et al., 2012; Guo et al., 2014; Chang et al., 2014; Huang et al., 2016). G-CSF is considered to be a neuronal ligand that acts through an autocrine protective mechanism which inhibits neuronal apoptosis by increasing the expression of anti-apoptotic genes or pro-survival signaling (Frank et al., 2009; Li et al., 2015; Huang et al., 2016). In this study, we found another mechanism which may explain the anti-inflammatory effects of G-CSF in the ONs in a rAION model. Through the dual actions of anti-apoptosis of RGCs and anti-inflammation at ON, early treatment with G-CSF within a therapeutic window provided neuroprotection in this rAION model.

### Conclusions

This study provides evidence for the mechanism by which early G-CSF treatment can have a neuroprotective effect in a rAION model. Early treatment with G-CSF can stabilize ON vascular permeability to reduce macrophage infiltration into the ON and induce M2 microglia/macrophage polarization at the ON. In addition, attenuation of macrophage infiltration and induction of M2 microglia/macrophage polarization can reduce pro-inflammatory cytokine expressions to prevent cytokine-induced apoptosis in a rAION model. Thus, early treatment with G-CSF (before 1 day post-infarct) can provide neuroprotective effects on RGCs and ONs, as well as preserving visual function.

## MATERIALS AND METHODS

### Study design

In the present study, we investigated the therapeutic window of G-CSF and the role of G-CSF in regulating ON inflammation in a rAION model (Fig. 1). After rAION had been induced, the rats were divided into six groups (*n*=18 in each group). Four groups received G-CSF treatment starting at different time points after infarction, including within 30 min after treatment (D0+G-CSF), and 24 h (D1+G-CSF), 48 h (D2+G-CSF) and 7 days (D7+G-CSF) post-rAION induction. One of the other two groups received subcutaneous injections of PBS post-infarct, and the other was a sham group. The treatment groups received a subcutaneous injection of G-CSF (100 μg/kg body weight/day in 0.2 ml of saline; Takasaki Pharmaceutical Plant, Tokyo, Japan) once daily for 5 days (Chang et al., 2014). At the 4th week post-infarct, RGC density was evaluated by retrograde labeling with FluoroGold in six rats of each group, immunohistochemistry and western blot in six rats of each group, and visual function was assessed by photoptic FVEP in the other six rats in each group. The rats were then euthanized by CO_2_ inhalation for tissue analysis (nine rats in each group). TUNEL assays of the RGC layer were performed. The newly synthesized (extrinsic) macrophages and monocytes (ED1) and microglial cell (Iba1) markers in the ON sections were investigated by immunohistochemistry. We compared the vascular permeability of the ON among groups to assess the intactness of the blood–ON barrier using Evans Blue extravasation and fluorescence post-rAION induction. Levels of the inflammatory cytokines TNFα and IL-1β were measured in the ON samples using western blotting analysis. The type of macrophage polarization was evaluated by real-time reverse-transcriptase (RT)-PCR.

### Animals

Adult male Wistar rats weighing 150-180 g (7- to 8-weeks old) were used in this study. The rats were obtained from the breeding colony of BioLASCO Co., Taiwan. Animal care and experimental procedures were conducted in accordance with the ARVO statement for the use of Animals in Ophthalmic and Vision Research, and the Institutional Animal Care and Use Committee (IACUC) at Tzu Chi General Hospital approved all of the animal experiments. All experiments were performed with the animals under general anesthesia induced by intramuscular injections of a mixture of ketamine (40 mg/kg body weight) and xylazine (4 mg/kg body weight; Sigma, St Louis, MO, USA), and topical 0.5% Alcaine eye drops (Alcon, Puurs, Belgium) (Huang et al., 2011; Chang et al., 2014). The rats had free access to food and water, and were maintained in cages in an environmentally controlled room with a temperature of 23±1°C, humidity of 55±5% and a 12-h light/dark cycle (light period: 7:00 a.m. to 7:00 p.m.).

### rAION

Details of the procedures have been reported in our study (Chang et al., 2014; Huang et al., 2015). Briefly, the ONHs of the rats were exposed to an argon laser immediately after an intravenous injection of the photosensitizing agent Rose Bengal (RB; Sigma-Aldrich, St Louis, MO, USA). In the rAION induction group, after general anesthesia, RB was injected intravenously through the tail vein. The right optic discs were directly exposed to an argon green laser 532-nm in wavelength, 500-μm in size and 80 mW in power (MC-500 Multi-color laser, Nidek Co., Ltd, Tokyo, Japan) after RB treatment. There were 12 pulses of 1 s duration for each laser procedure. The RB was activated by the green laser light to appear as a bright golden color, which was taken to indicate successful induction. In the sham group, the rats received sham laser treatment consisting of illuminating the ON region with an argon laser without RB administration. After laser treatment, Tobradex eye ointment (Alcon, Puurs, Belgium) was applied. Subsequently, the rats were kept on electric heating pads at 37°C and allowed to recover.

### Flash visual evoked potentials (FVEPs)

FVEPs were recorded 4 weeks after infarction. To avoid VEP responses of albino Wistar rats being contaminated from the contralateral side, we performed the same surgery in both eyes for FVEP examinations. This procedure was approved by the IACUC, and is detailed in our previous reports (Tsai et al., 2008; Chang et al., 2014; Huang et al., 2015). We used a visual electrodiagnostic system (UTAS-E3000, LKC Technologies, Gaithersburg, MD, USA) to measure photoptic FVEP. Previous studies had shown no significant differences in latency between photoptic and scotopic VEPs in Wistar rats, and a smaller amplitude in photoptic VEPs compared with the wave of scotopic VEPs (Heiduschka and Schraermeyer, 2008; Chang et al., 2014). We defined the first positive wavelet as the P1 wave (Young et al., 2012), and compared the latency of the P1 wave and the amplitude of the P1-N2 wave in each group (*n*=6 rats in each group) to evaluate visual function.

### Measurement of viable RGCs by retrograde labeling with FluoroGold

The procedure was performed as described in our previous reports (Tsai et al., 2008; Huang et al., 2011, 2015; Chang et al., 2014). Briefly, we performed retrograde labeling of the RGCs 1 week before the rats were euthanized to avoid over-counting the RGCs by mixing labeled RGCs with dye-engulfed macrophages and microglia (Huang et al., 2014). The skin covering the skull of the rats was opened, and 1.5 μl of 5% FluoroGold was injected into the superior colliculus on each side through a Hamilton syringe. One week after the labeling, the eyeballs were harvested after the animals had been euthanized (Huang et al., 2015). The RGCs of the retinas were examined at a distance of 1 or 3 mm from the center of the ONH to provide the central and mid-peripheral RGC densities, respectively. We counted five randomly chosen areas of 62,500 μm^2^ each in the central and mid-peripheral regions of each retina (*n*=6 per group). The survival rate of the RGCs (presented as a percentage) was calculated as the number of RGCs in each treatment group divided by the number of RGCs in the sham-operated group, and then multiplied by 100.

### ON and retinal sample preparation

#### ON preparation

A segment of the ON of about 5-7 mm in length behind the eyeball was collected after the rats had been sacrificed at 4 weeks. The nerves were immediately frozen at −70°C for histological and immunohistochemical (IHC) studies. None of the samples showed massive disc hemorrhage or retinal detachment on histological examination of the ONs (Huang et al., 2015).

#### Retinal section preparation

The cornea, lens and vitreous body were removed from the eyeball after the rats had been sacrificed. The remaining eyecups, containing the sclera and the retina, were fixed in 4% paraformaldehyde for 2 h at room temperature. Each retinal cup was cut adjacent to the disc into two pieces. The tissues were then dehydrated in 30% sucrose overnight and kept at −20°C until further processing. Some retinal cups were fixed in 4% paraformaldehyde for paraffin embedding and sectioning (Huang et al., 2015).

### *In situ* nick end-labeling (TUNEL) assay for apoptotic cell measurements

All frozen sections of the retinas were prepared with samples cut at 1-2 mm in distance from the ONH to ensure the use of equivalent fields for comparisons (Huang et al., 2011, 2015). TUNEL assays (DeadEnd™ Fluorometric TUNEL System, Promega Corporation, Madison, WI, USA) were performed to detect apoptotic cells. The TUNEL-positive cells in the RGC layer of each sample were averaged in ten HPFs (400× magnification).

### IHC of ED1- and Iba1-positive cells in the ONs

Anti-ED1 and -Iba1 antibodies react against extrinsic macrophages and intrinsic microglia, respectively, and we used monoclonal antibodies of anti-ED1 and -Iba1 (1:50, AbD Serotec, Oxford, UK) in this procedure. Described in a previous report (Chang et al., 2014), briefly, the frozen ON sections were fixed with acetone at −20°C for 30 min and then blocked with 5% fetal bovine serum containing 1% bovine serum albumin for 15 min. The primary antibody was applied and incubated overnight at 4°C. The secondary antibody conjugated with fluorescein isothiocyanate (FITC and Rhodamine, 1:100, Jackson ImmunoResearch Laboratories, West Grove, PA, USA) was incubated at room temperature for 1 h. Counterstaining was performed using 4′,6-diamidino-2-phenylindole (DAPI, 1:1000, Sigma, St Louis, MO, USA). For comparisons, the ED1-positive cells were counted in six HPFs (400× magnifications) at the ON lesion site.

### Vascular permeability of the ONs

The vascular permeability of the ONs was determined through Evans Blue extravasation as previously described (Scheppke et al., 2008; Xu et al., 2001). The rats were anesthetized with ketamine and xylazine, and 2% Evans Blue (Sigma-Aldrich, sonicated and filtered) in 0.9% NaCl was injected into the tail vein at a final dosage of 4 ml/kg body weight. The animals were then sacrificed 12 h later. A small amount of blood was collected via an intracardiac puncture. The ONs were dissected from the eyeball, allowed to dry, and then weighed. Half of the ON samples were used to analyze Evans Blue extravasation, and the other half were used to observe Evans Blue labeling under a fluorescence microscope. The samples were collected every day from day 0 to 4 post-rAION induction. Evans Blue dye conjugated to serum albumin in the ONs was extracted by solubilization in formamide (0.2 ml per retina) at 78°C overnight. The resulting suspensions were placed in an ultracentrifuge at 4°C at 128,000 ***g*** for 45 min. Evans Blue dye in the supernatant was detected spectrophotometrically by absorbance at 620 nm (blue signal) and 740 nm (subtracted background) and measured in comparison with a standard curve. Blood samples were treated similarly, but without solubilization and with centrifugation for 15 min at 3550 ***g*** at 25°C and at 1:1000 dilution prior to spectrometric evaluation. Evans Blue leakage was assessed by measuring ON dye accumulation using the following modified equation (Xu et al., 2001):
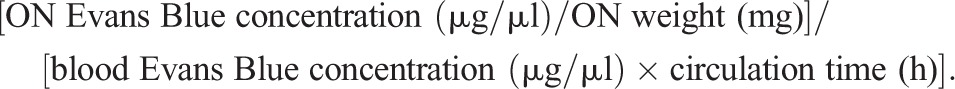


Other ON samples were fixed in 4% paraformaldehyde for paraffin embedding and sectioning. The location of the extravasated Evans Blue on the ON sections was observed with a fluorescence microscope at an excitation wavelength of 530-550 nm and emission wavelength of >590 nm as previous described (Rakos et al., 2007).

### Real-time RT-PCR of the M1/M2 macrophage markers

Arg1, CD206 and Fizz1 are markers of M2 macrophages, and CD32 and CD86 are markers of M1 macrophages (Orihuela et al., 2016). Tissue RNA was extracted using a Qiagen RNeasy Mini Kit from ON lysates obtained by sonication. All RNA samples were reverse transcribed for 30 min at 42°C with a High Capacity cDNA Reverse Transcription Kit according to the manufacturer's instructions (Applied Biosystems, Foster City, CA, USA). Real-time RT-PCR was conducted on an AB PRISM 7300 Sequence Detection System (Applied Biosystems). A QuantiTect SYBR green RT-PCR kit (Qiagen) was used. Expression levels of each test gene were normalized to those of *GypA* mRNA. Data were presented as the mean relative expression levels (±s.d.). The forward and reverse primers used in this study for amplification of the target genes were as follows (5′-3′): Arg1-F-TCGGAGCGCCTTTCTCTAAG; Arg1-R-ATCCCCGTGGTCTCTCACAT; CD206-F-AACGTTCGCTGATGCAAACC; CD206-R-TGTAAACTGCACCTGCTCGT; Fizz1-F-CAACAGGATGAAGACTGCAACCT; Fizz1-R-GGGACCATCAGCTAAAGAAG; CD86-F-AGACATGTGTAACCTGCACCAT; CD86-R-ACCGACTTTTTCCGGTCCTG; CD32-F-CTGTCGTCCATGTGCTCTCA; CD32-R-CAAGTTTCACCACAGCCTTCG; GypA-F-CACCGTGTTCTTCGACATCAC; GypA-R-CCAGTGCTCAGAGCACGAAAG.

### Western blotting

The ON samples were collected at day 7 post-rAION induction in each group. Western blotting was used to measure the levels of TNF-α and IL-1β in the ON samples. The ON protein extracts were separated using a 4-12% NuPAGE Bis-Tris gel (Invitrogen, Carlsbad, CA, USA). The separated proteins were then transferred onto polyvinylidene difluoride membranes and blocked with 5% of nonfat milk in Tris buffer saline/Tween-20 solution containing 20 mM Tris-HCl (pH 7.5), 0.5 M NaCl and 0.5% Tween-20. The membranes were then reacted with mouse anti-TNF-α antibodies, mouse anti-IL-1β antibodies, and goat anti-mouse immunoglobulin (Abcam, Cambridge, MA, USA). The blots were then developed using Enhanced Chemiluminescent Substrate (Perkin-Elmer Life Science, Boston, MA, USA), and the relative intensities of the bands were measured using an image analysis system (Amersham Biosciences Uppsala, Sweden).

### Statistical analysis

All statistical analyses were performed using a commercially available statistical software package (SPSS for Windows, version 19.0, SPSS, Chicago, IL, USA). The data were presented as mean±s.d. For comparisons of differences among groups, the non-parametric Mann–Whitney *U*-test for un-paired samples was applied. All *P*-values less than 0.05 were considered to be statistically significant.
